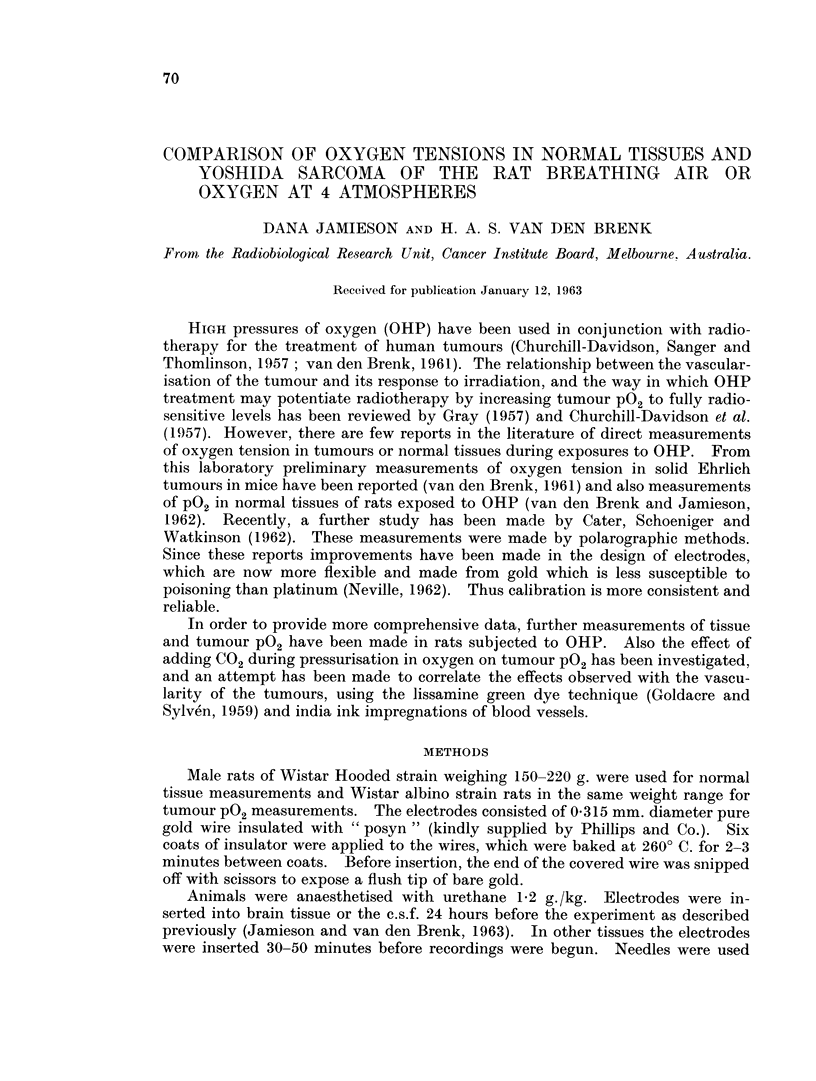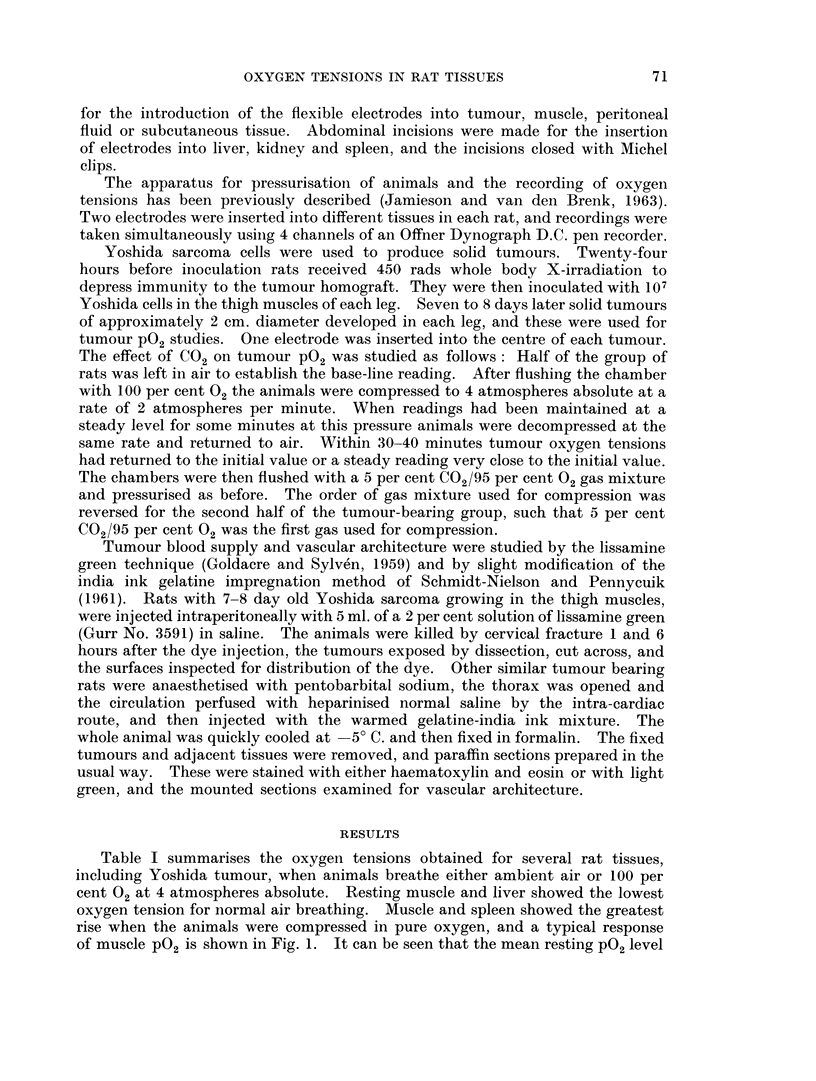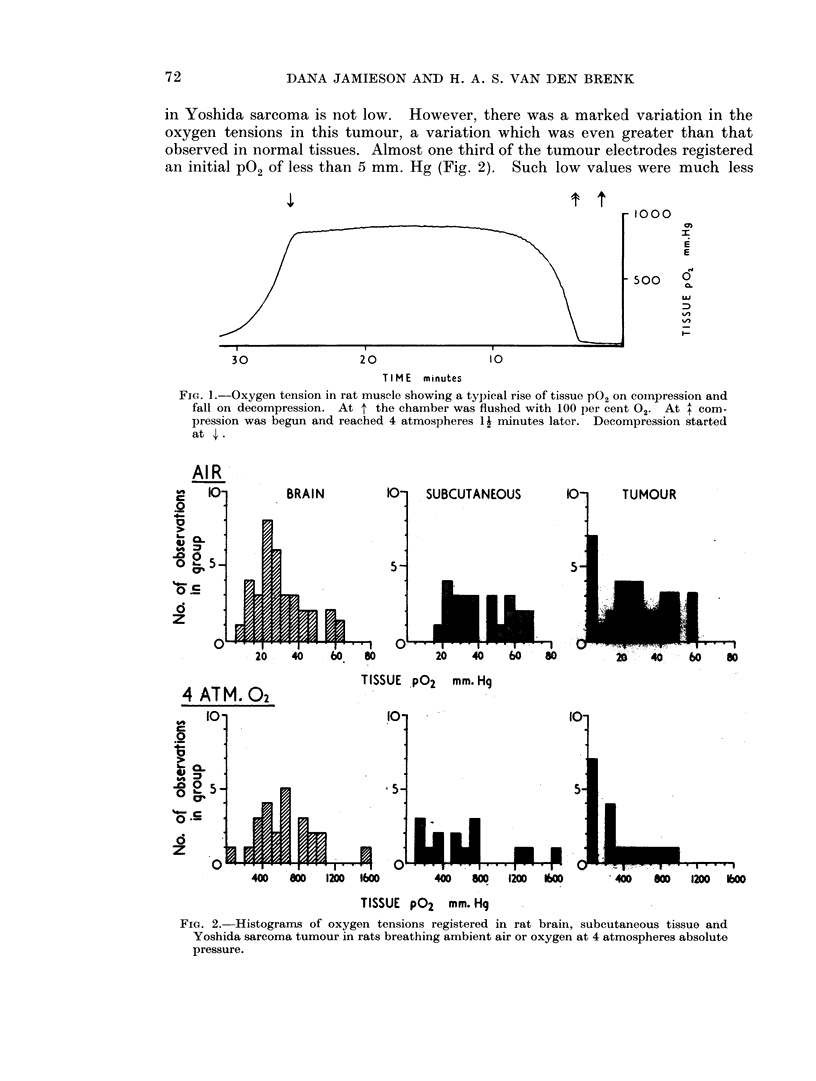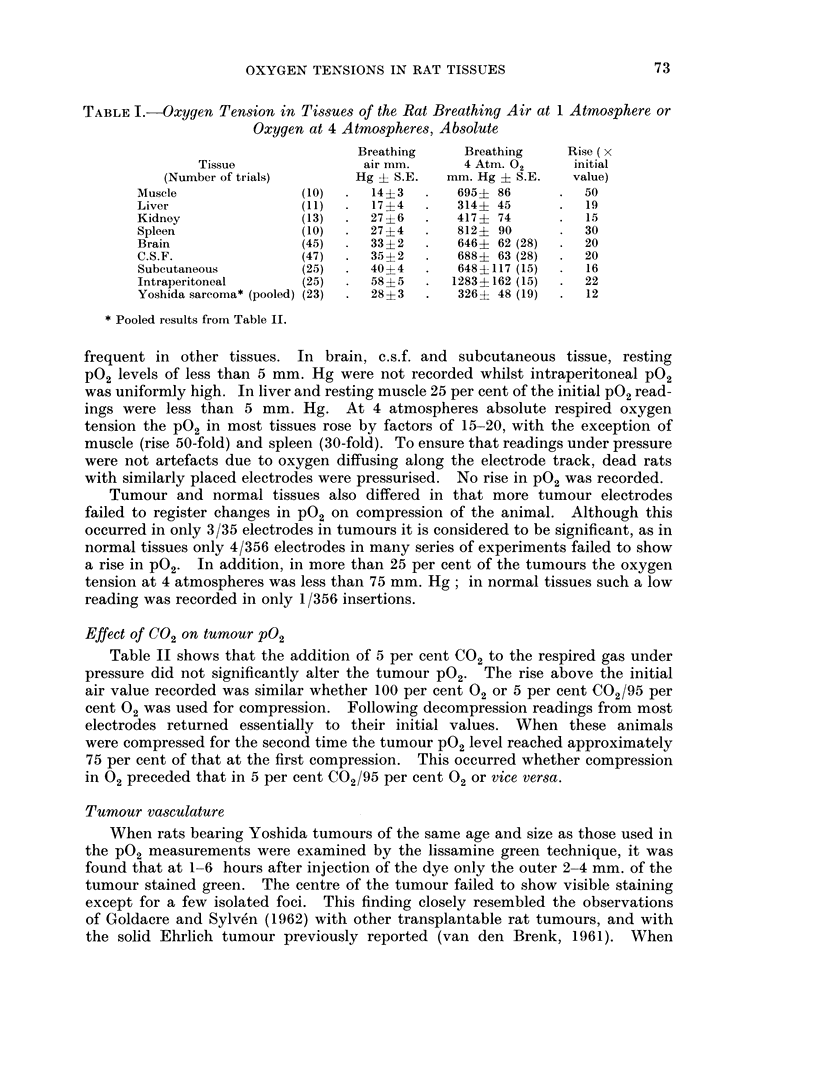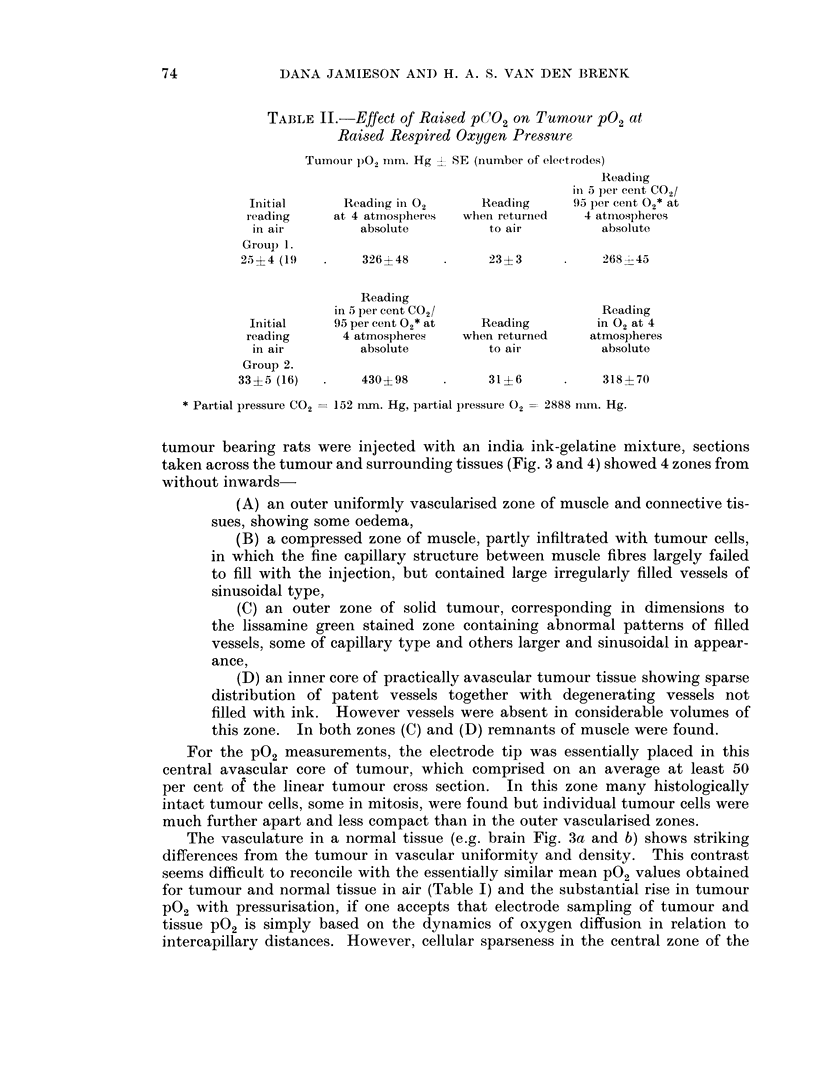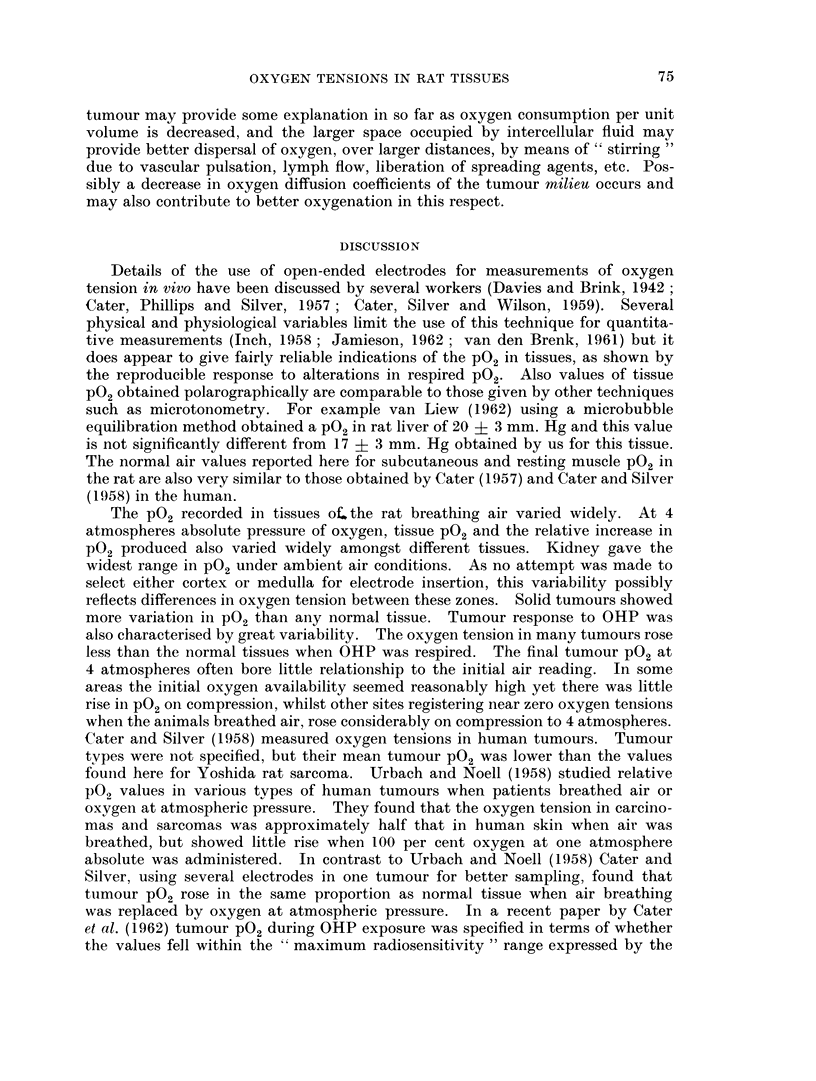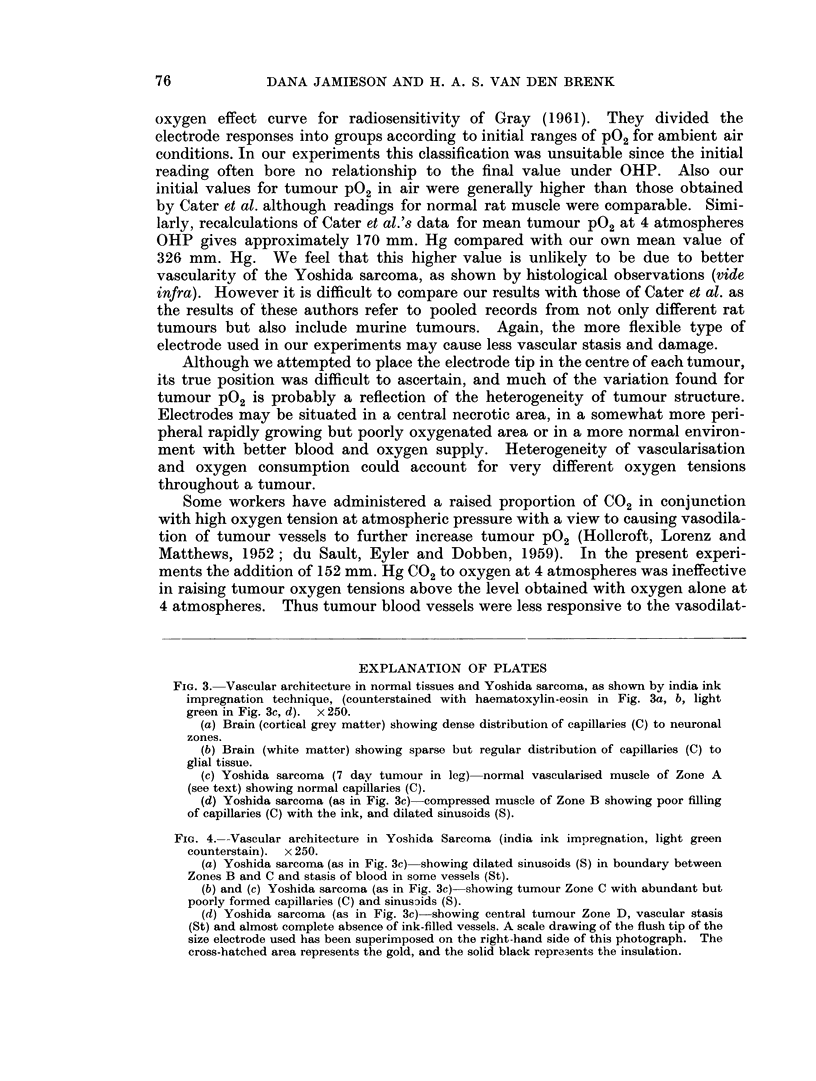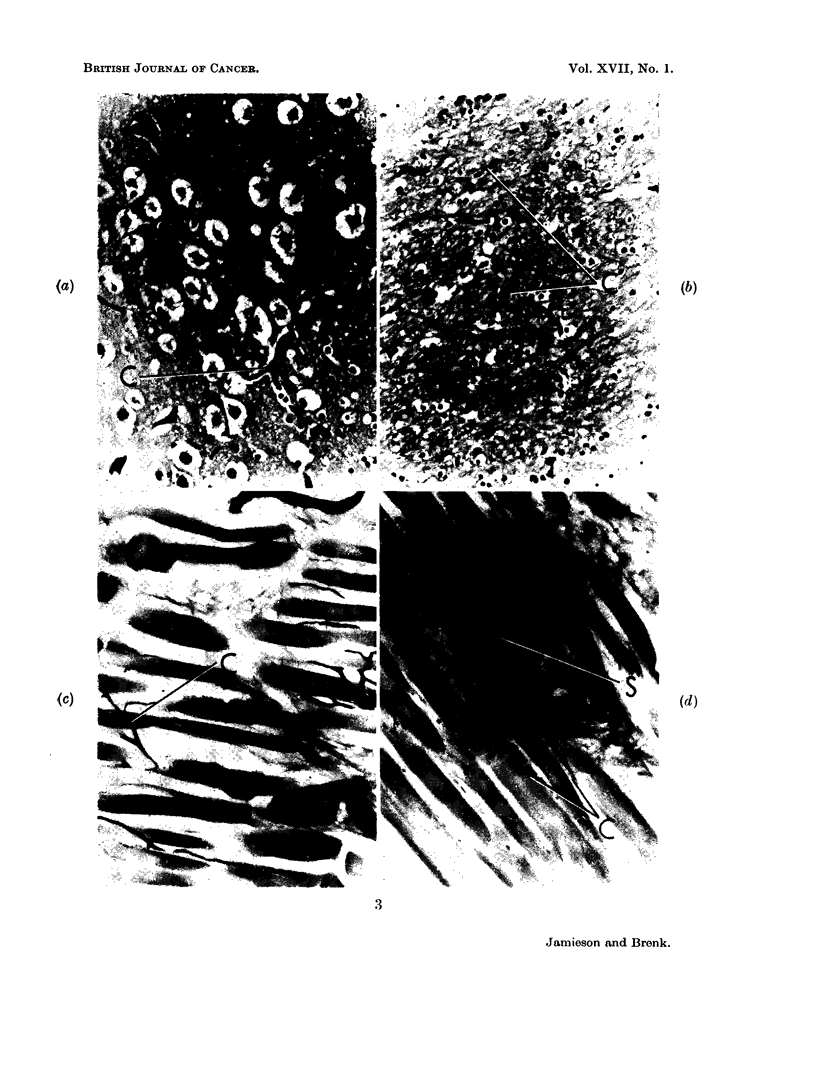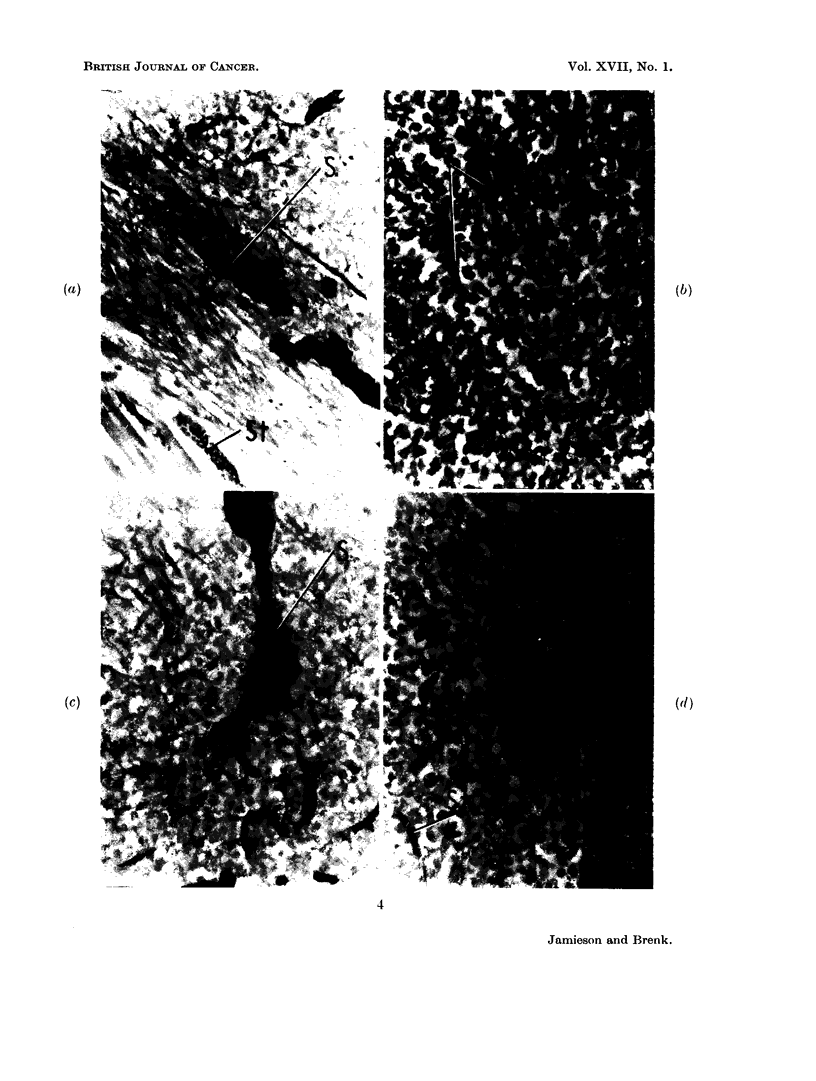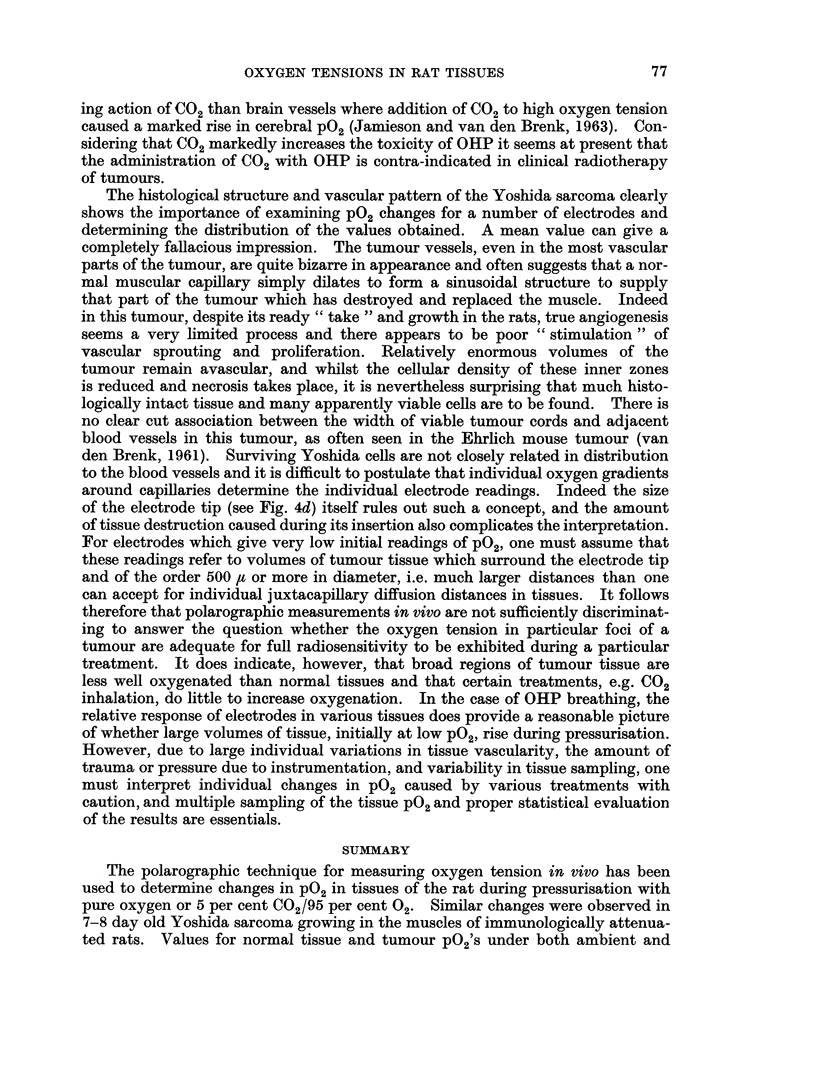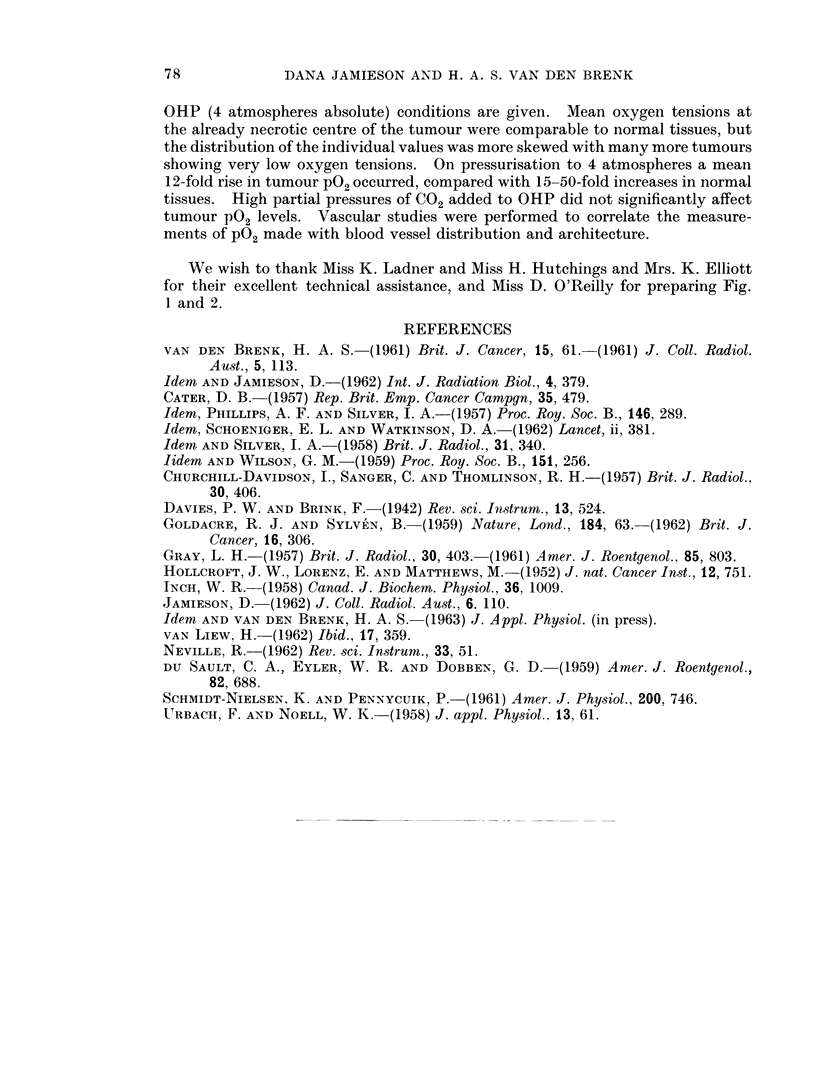# Comparison of Oxygen Tensions in Normal Tissues and Yoshida Sarcoma of the Rat Breathing Air or Oxygen at 4 Atmospheres

**DOI:** 10.1038/bjc.1963.11

**Published:** 1963-03

**Authors:** Dana Jamieson, H. A. S. van den Brenk

## Abstract

**Images:**


### COMPARISON OF OXYGEN TENSIONS IN NORMAL TISSUES AND YOSHIDA SARCOMA OF THE RAT BREATHING AIR OR OXYGEN AT 4 ATMOSPHERES

DANA JAMIESON AND H. A. S. VAN DEN BRENK

*From the Radiobiological Research Unit, Cancer Institute Board, Melbourne, Australia.*

Received for publication January 12, 1963

HIGH pressures of oxygen (OHP) have been used in conjunction with radiotherapy for the treatment of human tumours (Churchill-Davidson, Sanger and Thomlinson, 1957 ; van den Brenk, 1961). The relationship between the vascularisation of the tumour and its response to irradiation, and the way in which OHP treatment may potentiate radiotherapy by increasing tumour $pO_2$ to fully radiosensitive levels has been reviewed by Gray (1957) and Churchill-Davidson *et al.* (1957). However, there are few reports in the literature of direct measurements of oxygen tension in tumours or normal tissues during exposures to OHP. From this laboratory preliminary measurements of oxygen tension in solid Ehrlich tumours in mice have been reported (van den Brenk, 1961) and also measurements of $pO_2$ in normal tissues of rats exposed to OHP (van den Brenk and Jamieson, 1962). Recently, a further study has been made by Cater, Schoeniger and Watkinson (1962). These measurements were made by polarographic methods. Since these reports improvements have been made in the design of electrodes, which are now more flexible and made from gold which is less susceptible to poisoning than platinum (Neville, 1962). Thus calibration is more consistent and reliable.

In order to provide more comprehensive data, further measurements of tissue and tumour $pO_2$ have been made in rats subjected to OHP. Also the effect of adding $CO_2$ during pressurisation in oxygen on tumour $pO_2$ has been investigated, and an attempt has been made to correlate the effects observed with the vascularity of the tumours, using the lissamine green dye technique (Goldacre and Sylvén, 1959) and india ink impregnations of blood vessels.

### METHODS

Male rats of Wistar Hooded strain weighing 150–220 g. were used for normal tissue measurements and Wistar albino strain rats in the same weight range for tumour $pO_2$ measurements. The electrodes consisted of 0·315 mm. diameter pure gold wire insulated with " posyn " (kindly supplied by Phillips and Co.). Six coats of insulator were applied to the wires, which were baked at 260° C. for 2–3 minutes between coats. Before insertion, the end of the covered wire was snipped off with scissors to expose a flush tip of bare gold.

Animals were anaesthetised with urethane 1·2 g./kg. Electrodes were inserted into brain tissue or the c.s.f. 24 hours before the experiment as described previously (Jamieson and van den Brenk, 1963). In other tissues the electrodes were inserted 30–50 minutes before recordings were begun. Needles were used for the introduction of the flexible electrodes into tumour, muscle, peritoneal fluid or subcutaneous tissue. Abdominal incisions were made for the insertion of electrodes into liver, kidney and spleen, and the incisions closed with Michel clips.

The apparatus for pressurisation of animals and the recording of oxygen tensions has been previously described (Jamieson and van den Brenk, 1963). Two electrodes were inserted into different tissues in each rat, and recordings were taken simultaneously using 4 channels of an Offner Dynograph D.C. pen recorder.

Yoshida sarcoma cells were used to produce solid tumours. Twenty-four hours before inoculation rats received 450 rads whole body X-irradiation to depress immunity to the tumour homograft. They were then inoculated with $10^7$ Yoshida cells in the thigh muscles of each leg. Seven to 8 days later solid tumours of approximately 2 cm. diameter developed in each leg, and these were used for tumour $pO_2$ studies. One electrode was inserted into the centre of each tumour. The effect of $CO_2$ on tumour $pO_2$ was studied as follows : Half of the group of rats was left in air to establish the base-line reading. After flushing the chamber with 100 per cent $O_2$ the animals were compressed to 4 atmospheres absolute at a rate of 2 atmospheres per minute. When readings had been maintained at a steady level for some minutes at this pressure animals were decompressed at the same rate and returned to air. Within 30–40 minutes tumour oxygen tensions had returned to the initial value or a steady reading very close to the initial value. The chambers were then flushed with a 5 per cent $CO_2$/95 per cent $O_2$ gas mixture and pressurised as before. The order of gas mixture used for compression was reversed for the second half of the tumour-bearing group, such that 5 per cent $CO_2$/95 per cent $O_2$ was the first gas used for compression.

Tumour blood supply and vascular architecture were studied by the lissamine green technique (Goldacre and Sylvén, 1959) and by slight modification of the india ink gelatine impregnation method of Schmidt-Nielson and Pennycuik (1961). Rats with 7–8 day old Yoshida sarcoma growing in the thigh muscles, were injected intraperitoneally with 5 ml. of a 2 per cent solution of lissamine green (Gurr No. 3591) in saline. The animals were killed by cervical fracture 1 and 6 hours after the dye injection, the tumours exposed by dissection, cut across, and the surfaces inspected for distribution of the dye. Other similar tumour bearing rats were anaesthetised with pentobarbital sodium, the thorax was opened and the circulation perfused with heparinised normal saline by the intra-cardiac route, and then injected with the warmed gelatine-india ink mixture. The whole animal was quickly cooled at —5° C. and then fixed in formalin. The fixed tumours and adjacent tissues were removed, and paraffin sections prepared in the usual way. These were stained with either haematoxylin and eosin or with light green, and the mounted sections examined for vascular architecture.

### RESULTS

Table I summarises the oxygen tensions obtained for several rat tissues, including Yoshida tumour, when animals breathe either ambient air or 100 per cent $O_2$ at 4 atmospheres absolute. Resting muscle and liver showed the lowest oxygen tension for normal air breathing. Muscle and spleen showed the greatest rise when the animals were compressed in pure oxygen, and a typical response of muscle $pO_2$ is shown in Fig. 1. It can be seen that the mean resting $pO_2$ level in Yoshida sarcoma is not low. However, there was a marked variation in the oxygen tensions in this tumour, a variation which was even greater than that observed in normal tissues. Almost one third of the tumour electrodes registered an initial $pO_2$ of less than 5 mm. Hg (Fig. 2). Such low values were much less

FIG. 1.—Oxygen tension in rat muscle showing a typical rise of tissue $pO_2$ on compression and fall on decompression. At ↑ the chamber was flushed with 100 per cent $O_2$. At ↟ compression was begun and reached 4 atmospheres 1½ minutes later. Decompression started at ↓.

FIG. 2.—Histograms of oxygen tensions registered in rat brain, subcutaneous tissue and Yoshida sarcoma tumour in rats breathing ambient air or oxygen at 4 atmospheres absolute pressure.

TABLE I.—*Oxygen Tension in Tissues of the Rat Breathing Air at 1 Atmosphere or Oxygen at 4 Atmospheres, Absolute*

| Tissue (Number of trials) | | Breathing air mm. Hg ± S.E. | Breathing 4 Atm. $O_2$ mm. Hg ± S.E. | Rise (× initial value) |
|---|---|---|---|---|
| Muscle | (10) | 14±3 | 695± 86 | 50 |
| Liver | (11) | 17±4 | 314± 45 | 19 |
| Kidney | (13) | 27±6 | 417± 74 | 15 |
| Spleen | (10) | 27±4 | 812± 90 | 30 |
| Brain | (45) | 33±2 | 646± 62 (28) | 20 |
| C.S.F. | (47) | 35±2 | 688± 63 (28) | 20 |
| Subcutaneous | (25) | 40±4 | 648±117 (15) | 16 |
| Intraperitoneal | (25) | 58±5 | 1283±162 (15) | 22 |
| Yoshida sarcoma* (pooled) | (23) | 28±3 | 326± 48 (19) | 12 |

* Pooled results from Table II.

frequent in other tissues. In brain, c.s.f. and subcutaneous tissue, resting $pO_2$ levels of less than 5 mm. Hg were not recorded whilst intraperitoneal $pO_2$ was uniformly high. In liver and resting muscle 25 per cent of the initial $pO_2$ readings were less than 5 mm. Hg. At 4 atmospheres absolute respired oxygen tension the $pO_2$ in most tissues rose by factors of 15–20, with the exception of muscle (rise 50-fold) and spleen (30-fold). To ensure that readings under pressure were not artefacts due to oxygen diffusing along the electrode track, dead rats with similarly placed electrodes were pressurised. No rise in $pO_2$ was recorded.

Tumour and normal tissues also differed in that more tumour electrodes failed to register changes in $pO_2$ on compression of the animal. Although this occurred in only 3/35 electrodes in tumours it is considered to be significant, as in normal tissues only 4/356 electrodes in many series of experiments failed to show a rise in $pO_2$. In addition, in more than 25 per cent of the tumours the oxygen tension at 4 atmospheres was less than 75 mm. Hg ; in normal tissues such a low reading was recorded in only 1/356 insertions.

*Effect of $CO_2$ on tumour $pO_2$*

Table II shows that the addition of 5 per cent $CO_2$ to the respired gas under pressure did not significantly alter the tumour $pO_2$. The rise above the initial air value recorded was similar whether 100 per cent $O_2$ or 5 per cent $CO_2$/95 per cent $O_2$ was used for compression. Following decompression readings from most electrodes returned essentially to their initial values. When these animals were compressed for the second time the tumour $pO_2$ level reached approximately 75 per cent of that at the first compression. This occurred whether compression in $O_2$ preceded that in 5 per cent $CO_2$/95 per cent $O_2$ or *vice versa.*

*Tumour vasculature*

When rats bearing Yoshida tumours of the same age and size as those used in the $pO_2$ measurements were examined by the lissamine green technique, it was found that at 1–6 hours after injection of the dye only the outer 2–4 mm. of the tumour stained green. The centre of the tumour failed to show visible staining except for a few isolated foci. This finding closely resembled the observations of Goldacre and Sylvén (1962) with other transplantable rat tumours, and with the solid Ehrlich tumour previously reported (van den Brenk, 1961). When

TABLE II.—*Effect of Raised* $pCO_2$ *on Tumour* $pO_2$ *at Raised Respired Oxygen Pressure*

Tumour $pO_2$ mm. Hg ± SE (number of electrodes)

| Initial reading in air | Reading in $O_2$ at 4 atmospheres absolute | Reading when returned to air | Reading in 5 per cent $CO_2$/95 per cent $O_2$* at 4 atmospheres absolute |
|---|---|---|---|
| Group 1. | | | |
| 25±4 (19 | 326±48 | 23±3 | 268±45 |

| Initial reading in air | Reading in 5 per cent $CO_2$/95 per cent $O_2$* at 4 atmospheres absolute | Reading when returned to air | Reading in $O_2$ at 4 atmospheres absolute |
|---|---|---|---|
| Group 2. | | | |
| 33±5 (16) | 430±98 | 31±6 | 318±70 |

* Partial pressure $CO_2$ = 152 mm. Hg, partial pressure $O_2$ = 2888 mm. Hg.

tumour bearing rats were injected with an india ink-gelatine mixture, sections taken across the tumour and surrounding tissues (Fig. 3 and 4) showed 4 zones from without inwards—

(A) an outer uniformly vascularised zone of muscle and connective tissues, showing some oedema,

(B) a compressed zone of muscle, partly infiltrated with tumour cells, in which the fine capillary structure between muscle fibres largely failed to fill with the injection, but contained large irregularly filled vessels of sinusoidal type,

(C) an outer zone of solid tumour, corresponding in dimensions to the lissamine green stained zone containing abnormal patterns of filled vessels, some of capillary type and others larger and sinusoidal in appearance,

(D) an inner core of practically avascular tumour tissue showing sparse distribution of patent vessels together with degenerating vessels not filled with ink. However vessels were absent in considerable volumes of this zone. In both zones (C) and (D) remnants of muscle were found.

For the $pO_2$ measurements, the electrode tip was essentially placed in this central avascular core of tumour, which comprised on an average at least 50 per cent of the linear tumour cross section. In this zone many histologically intact tumour cells, some in mitosis, were found but individual tumour cells were much further apart and less compact than in the outer vascularised zones.

The vasculature in a normal tissue (e.g. brain Fig. 3*a* and *b*) shows striking differences from the tumour in vascular uniformity and density. This contrast seems difficult to reconcile with the essentially similar mean $pO_2$ values obtained for tumour and normal tissue in air (Table I) and the substantial rise in tumour $pO_2$ with pressurisation, if one accepts that electrode sampling of tumour and tissue $pO_2$ is simply based on the dynamics of oxygen diffusion in relation to intercapillary distances. However, cellular sparseness in the central zone of the tumour may provide some explanation in so far as oxygen consumption per unit volume is decreased, and the larger space occupied by intercellular fluid may provide better dispersal of oxygen, over larger distances, by means of " stirring " due to vascular pulsation, lymph flow, liberation of spreading agents, etc. Possibly a decrease in oxygen diffusion coefficients of the tumour *milieu* occurs and may also contribute to better oxygenation in this respect.

### DISCUSSION

Details of the use of open-ended electrodes for measurements of oxygen tension *in vivo* have been discussed by several workers (Davies and Brink, 1942 ; Cater, Phillips and Silver, 1957 ; Cater, Silver and Wilson, 1959). Several physical and physiological variables limit the use of this technique for quantitative measurements (Inch, 1958 ; Jamieson, 1962 ; van den Brenk, 1961) but it does appear to give fairly reliable indications of the $pO_2$ in tissues, as shown by the reproducible response to alterations in respired $pO_2$. Also values of tissue $pO_2$ obtained polarographically are comparable to those given by other techniques such as microtonometry. For example van Liew (1962) using a microbubble equilibration method obtained a $pO_2$ in rat liver of $20 \pm 3$ mm. Hg and this value is not significantly different from $17 \pm 3$ mm. Hg obtained by us for this tissue. The normal air values reported here for subcutaneous and resting muscle $pO_2$ in the rat are also very similar to those obtained by Cater (1957) and Cater and Silver (1958) in the human.

The $pO_2$ recorded in tissues of the rat breathing air varied widely. At 4 atmospheres absolute pressure of oxygen, tissue $pO_2$ and the relative increase in $pO_2$ produced also varied widely amongst different tissues. Kidney gave the widest range in $pO_2$ under ambient air conditions. As no attempt was made to select either cortex or medulla for electrode insertion, this variability possibly reflects differences in oxygen tension between these zones. Solid tumours showed more variation in $pO_2$ than any normal tissue. Tumour response to OHP was also characterised by great variability. The oxygen tension in many tumours rose less than the normal tissues when OHP was respired. The final tumour $pO_2$ at 4 atmospheres often bore little relationship to the initial air reading. In some areas the initial oxygen availability seemed reasonably high yet there was little rise in $pO_2$ on compression, whilst other sites registering near zero oxygen tensions when the animals breathed air, rose considerably on compression to 4 atmospheres. Cater and Silver (1958) measured oxygen tensions in human tumours. Tumour types were not specified, but their mean tumour $pO_2$ was lower than the values found here for Yoshida rat sarcoma. Urbach and Noell (1958) studied relative $pO_2$ values in various types of human tumours when patients breathed air or oxygen at atmospheric pressure. They found that the oxygen tension in carcinomas and sarcomas was approximately half that in human skin when air was breathed, but showed little rise when 100 per cent oxygen at one atmosphere absolute was administered. In contrast to Urbach and Noell (1958) Cater and Silver, using several electrodes in one tumour for better sampling, found that tumour $pO_2$ rose in the same proportion as normal tissue when air breathing was replaced by oxygen at atmospheric pressure. In a recent paper by Cater *et al.* (1962) tumour $pO_2$ during OHP exposure was specified in terms of whether the values fell within the " maximum radiosensitivity " range expressed by the oxygen effect curve for radiosensitivity of Gray (1961). They divided the electrode responses into groups according to initial ranges of $pO_2$ for ambient air conditions. In our experiments this classification was unsuitable since the initial reading often bore no relationship to the final value under OHP. Also our initial values for tumour $pO_2$ in air were generally higher than those obtained by Cater *et al.* although readings for normal rat muscle were comparable. Similarly, recalculations of Cater *et al.'s* data for mean tumour $pO_2$ at 4 atmospheres OHP gives approximately 170 mm. Hg compared with our own mean value of 326 mm. Hg. We feel that this higher value is unlikely to be due to better vascularity of the Yoshida sarcoma, as shown by histological observations (*vide infra*). However it is difficult to compare our results with those of Cater *et al.* as the results of these authors refer to pooled records from not only different rat tumours but also include murine tumours. Again, the more flexible type of electrode used in our experiments may cause less vascular stasis and damage.

Although we attempted to place the electrode tip in the centre of each tumour, its true position was difficult to ascertain, and much of the variation found for tumour $pO_2$ is probably a reflection of the heterogeneity of tumour structure. Electrodes may be situated in a central necrotic area, in a somewhat more peripheral rapidly growing but poorly oxygenated area or in a more normal environment with better blood and oxygen supply. Heterogeneity of vascularisation and oxygen consumption could account for very different oxygen tensions throughout a tumour.

Some workers have administered a raised proportion of $CO_2$ in conjunction with high oxygen tension at atmospheric pressure with a view to causing vasodilation of tumour vessels to further increase tumour $pO_2$ (Hollcroft, Lorenz and Matthews, 1952; du Sault, Eyler and Dobben, 1959). In the present experiments the addition of 152 mm. Hg $CO_2$ to oxygen at 4 atmospheres was ineffective in raising tumour oxygen tensions above the level obtained with oxygen alone at 4 atmospheres. Thus tumour blood vessels were less responsive to the vasodilat-

---

EXPLANATION OF PLATES

FIG. 3.—Vascular architecture in normal tissues and Yoshida sarcoma, as shown by india ink impregnation technique, (counterstained with haematoxylin-eosin in Fig. 3*a*, *b*, light green in Fig. 3*c*, *d*). ×250.

(*a*) Brain (cortical grey matter) showing dense distribution of capillaries (C) to neuronal zones.

(*b*) Brain (white matter) showing sparse but regular distribution of capillaries (C) to glial tissue.

(*c*) Yoshida sarcoma (7 day tumour in leg)—normal vascularised muscle of Zone A (see text) showing normal capillaries (C).

(*d*) Yoshida sarcoma (as in Fig. 3*c*)—compressed muscle of Zone B showing poor filling of capillaries (C) with the ink, and dilated sinusoids (S).

FIG. 4.—Vascular architecture in Yoshida Sarcoma (india ink impregnation, light green counterstain). ×250.

(*a*) Yoshida sarcoma (as in Fig. 3*c*)—showing dilated sinusoids (S) in boundary between Zones B and C and stasis of blood in some vessels (St).

(*b*) and (*c*) Yoshida sarcoma (as in Fig. 3*c*)—showing tumour Zone C with abundant but poorly formed capillaries (C) and sinusoids (S).

(*d*) Yoshida sarcoma (as in Fig. 3*c*)—showing central tumour Zone D, vascular stasis (St) and almost complete absence of ink-filled vessels. A scale drawing of the flush tip of the size electrode used has been superimposed on the right-hand side of this photograph. The cross-hatched area represents the gold, and the solid black represents the insulation.

3

Jamieson and Brenk.

(a) (b)

(c) (d)

4

ing action of $CO_2$ than brain vessels where addition of $CO_2$ to high oxygen tension caused a marked rise in cerebral $pO_2$ (Jamieson and van den Brenk, 1963). Considering that $CO_2$ markedly increases the toxicity of OHP it seems at present that the administration of $CO_2$ with OHP is contra-indicated in clinical radiotherapy of tumours.

The histological structure and vascular pattern of the Yoshida sarcoma clearly shows the importance of examining $pO_2$ changes for a number of electrodes and determining the distribution of the values obtained. A mean value can give a completely fallacious impression. The tumour vessels, even in the most vascular parts of the tumour, are quite bizarre in appearance and often suggests that a normal muscular capillary simply dilates to form a sinusoidal structure to supply that part of the tumour which has destroyed and replaced the muscle. Indeed in this tumour, despite its ready " take " and growth in the rats, true angiogenesis seems a very limited process and there appears to be poor " stimulation " of vascular sprouting and proliferation. Relatively enormous volumes of the tumour remain avascular, and whilst the cellular density of these inner zones is reduced and necrosis takes place, it is nevertheless surprising that much histologically intact tissue and many apparently viable cells are to be found. There is no clear cut association between the width of viable tumour cords and adjacent blood vessels in this tumour, as often seen in the Ehrlich mouse tumour (van den Brenk, 1961). Surviving Yoshida cells are not closely related in distribution to the blood vessels and it is difficult to postulate that individual oxygen gradients around capillaries determine the individual electrode readings. Indeed the size of the electrode tip (see Fig. 4*d*) itself rules out such a concept, and the amount of tissue destruction caused during its insertion also complicates the interpretation. For electrodes which give very low initial readings of $pO_2$, one must assume that these readings refer to volumes of tumour tissue which surround the electrode tip and of the order 500 $\mu$ or more in diameter, i.e. much larger distances than one can accept for individual juxtacapillary diffusion distances in tissues. It follows therefore that polarographic measurements *in vivo* are not sufficiently discriminating to answer the question whether the oxygen tension in particular foci of a tumour are adequate for full radiosensitivity to be exhibited during a particular treatment. It does indicate, however, that broad regions of tumour tissue are less well oxygenated than normal tissues and that certain treatments, e.g. $CO_2$ inhalation, do little to increase oxygenation. In the case of OHP breathing, the relative response of electrodes in various tissues does provide a reasonable picture of whether large volumes of tissue, initially at low $pO_2$, rise during pressurisation. However, due to large individual variations in tissue vascularity, the amount of trauma or pressure due to instrumentation, and variability in tissue sampling, one must interpret individual changes in $pO_2$ caused by various treatments with caution, and multiple sampling of the tissue $pO_2$ and proper statistical evaluation of the results are essentials.

### SUMMARY

The polarographic technique for measuring oxygen tension *in vivo* has been used to determine changes in $pO_2$ in tissues of the rat during pressurisation with pure oxygen or 5 per cent $CO_2$/95 per cent $O_2$. Similar changes were observed in 7–8 day old Yoshida sarcoma growing in the muscles of immunologically attenuated rats. Values for normal tissue and tumour $pO_2$'s under both ambient and

OHP (4 atmospheres absolute) conditions are given. Mean oxygen tensions at the already necrotic centre of the tumour were comparable to normal tissues, but the distribution of the individual values was more skewed with many more tumours showing very low oxygen tensions. On pressurisation to 4 atmospheres a mean 12-fold rise in tumour $pO_2$ occurred, compared with 15–50-fold increases in normal tissues. High partial pressures of $CO_2$ added to OHP did not significantly affect tumour $pO_2$ levels. Vascular studies were performed to correlate the measurements of $pO_2$ made with blood vessel distribution and architecture.

We wish to thank Miss K. Ladner and Miss H. Hutchings and Mrs. K. Elliott for their excellent technical assistance, and Miss D. O'Reilly for preparing Fig. 1 and 2.

### REFERENCES

van den Brenk, H. A. S.—(1961) *Brit. J. Cancer*, **15**, 61.—(1961) *J. Coll. Radiol. Aust.*, **5**, 113.
*Idem* and Jamieson, D.—(1962) *Int. J. Radiation Biol.*, **4**, 379.
Cater, D. B.—(1957) *Rep. Brit. Emp. Cancer Campgn*, **35**, 479.
*Idem*, Phillips, A. F. and Silver, I. A.—(1957) *Proc. Roy. Soc.* B., **146**, 289.
*Idem*, Schoeniger, E. L. and Watkinson, D. A.—(1962) *Lancet*, ii, 381.
*Idem* and Silver, I. A.—(1958) *Brit. J. Radiol.*, **31**, 340.
*Iidem* and Wilson, G. M.—(1959) *Proc. Roy. Soc.* B., **151**, 256.
Churchill-Davidson, I., Sanger, C. and Thomlinson, R. H.—(1957) *Brit. J. Radiol.*, **30**, 406.
Davies, P. W. and Brink, F.—(1942) *Rev. sci. Instrum.*, **13**, 524.
Goldacre, R. J. and Sylvén, B.—(1959) *Nature, Lond.*, **184**, 63.—(1962) *Brit. J. Cancer*, **16**, 306.
Gray, L. H.—(1957) *Brit. J. Radiol.*, **30**, 403.—(1961) *Amer. J. Roentgenol.*, **85**, 803.
Hollcroft, J. W., Lorenz, E. and Matthews, M.—(1952) *J. nat. Cancer Inst.*, **12**, 751.
Inch, W. R.—(1958) *Canad. J. Biochem. Physiol.*, **36**, 1009.
Jamieson, D.—(1962) *J. Coll. Radiol. Aust.*, **6**. 110.
*Idem* and van den Brenk, H. A. S.—(1963) *J. Appl. Physiol.* (in press).
van Liew, H.—(1962) *Ibid.*, **17**, 359.
Neville, R.—(1962) *Rev. sci. Instrum.*, **33**, 51.
du Sault, C. A., Eyler, W. R. and Dobben, G. D.—(1959) *Amer. J. Roentgenol.*, **82**, 688.
Schmidt-Nielsen, K. and Pennycuik, P.—(1961) *Amer. J. Physiol.*, **200**, 746.
Urbach, F. and Noell, W. K.—(1958) *J. appl. Physiol.*, **13**, 61.